# Asthma control conundrum in clinical practice – Data from a two-stage Delphi survey and literature review

**DOI:** 10.1016/j.dib.2023.109422

**Published:** 2023-07-18

**Authors:** Giorgio Walter Canonica, Antonio Spanevello, Luis Pérez de Llano, Christian Domingo Ribas, John D Blakey, Gabriel Garcia, Hiromasa Inoue, Margareth Dalcolmo, Dong Yang, Soniya Mokashi, Abhishek Kurne, Aman Kapil Butta

**Affiliations:** a Humanitas University, Personalized Medicine Asthma & Allergy Clinic-Humanitas Research Hospital IRCCS, Milan, Italy; b Dipartimento di Medicina e Chirurgia, Università degli Studi dell'Insubria, Varese, Italy, Dipartimento di Medicina e Riabilitazione Cardio-Respiratoria, Istituti Clinici Scientici Maugeri, IRCCS Tradate (VA), Italy; c Pneumology Service, Hospital Universitario Lucus Augusti, EOXI Lugo, Cervo e Monforte, Spain; d Pulmonary Service, Corporació Sanitària Parc Taulí. Universitat Autònoma de Barcelona (UAB)., Sabadell, Spain; e Respiratory Medicine, Sir Charles Gairdner Hospital, Nedlands, Western Australia, Australia; f Medical School, Curtin University, Perth, Australia; g Pneumology, Pulmonary Service, Hospital R Rossi, La Plata, Argentina; h Department of Pulmonary Medicine, Graduate School of Medical and Dental Sciences, Kagoshima University, Kagoshima, Japan; i Reference Center Hélio Fraga, Fundação Oswaldo Cruz (Fiocruz)/Ministry of Health, Rio de Janeiro, Brazil; j Department of Pulmonary Medicine, Zhongshan Hospital, Fudan University, Shanghai, China; k GSK, Brentford, Middlesex, UK; l Global Medical Affairs, GSK, Asia House, Singapore; m GSK Asia House, Singapore

**Keywords:** Asthma expert recommendation, Quantitative form, Asthma communication, Asthma consensus, Screening questions, Standard definitions in asthma, Disparities in asthma control

## Abstract

Definitions and measures of asthma control used in clinical trials and practice often vary, as highlighted in the manuscript, “Is asthma control more than just an absence of symptoms? An expert consensus statement”. Furthermore, the authors discussed differences between patients and healthcare professionals (HCPs) in terms of understanding and managing asthma. Given these disparities, there is a need for consensus regarding what constitutes well-controlled asthma and, especially, how best it can be measured and recorded. In the current work, we describe our data and provide more detail on the methodology from a two-stage Delphi survey and a structured literature review, which were designed to reach a consensus definition of asthma control and alleviate misalignments between patients and HCPs. Survey data were collected using a two-stage Delphi technique; a method used to collate expert opinions over a series of sequential questionnaires to reach a consensus. The collated Delphi survey data were compared with results from a comprehensive, structured literature review of 216 publications, to assess if there was a correlation between existing guidance and measures of asthma control used in clinical trials and standard clinical practice. In order to collate and interpret findings from the Delphi survey, responses from 82 panelists (73 HCPs and 9 authors) were qualitatively analyzed, quantitatively categorized, and presented as percentages or counts in Excel databases, which are detailed in the current work. Searches conducted using PubMed and Cochrane identified 664 manuscripts, and Embase was used to identify 89 congress abstracts. After applying a stringent screening method using predefined key words, the structured literature review consisted of 185 peer-reviewed manuscripts and 31 congress abstracts, and assessed existing guidance and measures of asthma control used in clinical trials. In this publication, we provide further insight into the predefined keywords, search strings, and strategy applied to identify manuscripts and congress abstracts for inclusion/exclusion, and detail methods for data extraction. Together, the data from the Delphi survey and structured literature review aimed to provide greater insights into challenges and approaches in achieving asthma control in clinical practice, with the potential for results to be used to guide a universally accepted definition and measure of asthma control that can be used and understood by patients, HCPs, and researchers. Qualitative and quantitative methodology and analysis from the Delphi survey and literature review search strategy can potentially be used to identify disparities and explore expert opinion and relevant literature in other therapeutic areas to guide a consensus where disparities exist.

Specifications Table

**Table:** 

Subject	Pulmonary and Respiratory Medicine
Specific subject area	Understanding and managing asthma control in clinical trials to alleviate disparities between healthcare professionals (HCPs) and patient perspectives.
Type of data	Tables, Graphs, Figures
How the data were acquired	Expert HCPs (*n* = 82) participated in the Delphi survey [1]. Subsequent analysis was performed according to the two-stage Delphi technique. First-round of questions was analyzed to guide themes of questions for the second-round survey (questionnaires, selected literature publications, and abstracts from the literature review, are provided in the online repository). Publications and abstracts from the literature review were chosen based on a selection criterion using PubMed and Cochrane databases (search strings are provided in the online repository).
Data format	Raw data
Description of data collection	**Raw data are published on the Zenodo online repository website.** **Screening criteria for panelists in the two-stage Delphi survey** The panelists were identified through SERMO (a third-party, centralized database of HCPs). Screening criteria ensured that only those with an appropriate specialty (pulmonologists, allergists, and general practitioners), years of experience in their specialty (> 3 years), and proportion of time actively treating patients (≥ 40%) were included. Panelists who completed the questionnaire within the specified 1-week time frame were eligible to participate in the second-round questionnaire. Overall, 63 panelists, including nine of the authors, took part in the second-round Delphi questionnaire (19 panelists were lost in the first-round follow up). **Structured literature review** Publications and abstracts were selected based on the following criteria: study in asthma, patients aged above 18 years, randomized controlled trial (RCT) systematic review, meta-analysis, real-world evidence (RWE), observational, retrospective, cross-sectional, prospective, longitudinal, control as a study endpoint, novel data, English language. The exclusion criteria were as follows: incorrect/mixed indication, ineligible study design, ineligible patient population, outcomes of interest not reported, no novel data reported, excluded publication types missing data, not in English language, duplicates.
Data source location	An equal number of panelists were invited from Argentina, Australia, Brazil, China, Italy, Japan, and Spain. Panelists’ information was kept confidential and anonymous according to the UK Data Protection Act (GDPR) and with the British Healthcare Business Intelligence Association's (BHBIA) Legal & Ethical Guidelines, along with the European Pharmaceutical Market Research Association's (EphMRA).
Data accessibility	Link to Zenodo online repository: https://zenodo.org/record/8043569
Related research article	G.W. Canonica, A. Spanevello, L.P. de Llano, C.D. Ribas, J.D. Blakey, G. Garcia, H. Inoue, M. Dalcolmo, D. Yang, S. Mokashi, A. Kurne, A.K. Butta, 2022. Is asthma control more than just an absence of symptoms? An expert consensus statement. *Respir Med*, 202, p.106942.

## 1 Value of the Data

• Data derived from a two-stage Delphi survey involving asthma specialists consist of questions that sought to identify areas of consensus on aspects of asthma control in clinical practice. Together with the structured literature review, the data assessed if existing guidance and measures of asthma control used in studies correlated with practice.
• Data are aimed at progressing towards a consensus definition of asthma control and clarifying disparities between HCP and patient perspectives.
• The dataset has the potential to inform HCPs (pulmonologists, allergists, and general practitioners*)*, patients, and researchers from health settings to make informed decisions and provide understanding on the assessment of asthma.
• The structured literature search has the potential to provide more insight into asthma management and guidelines.

## 2 Objective

The data described in this article provides additional value to the manuscript, “Is asthma control more than just an absence of symptoms? An expert consensus statement”, by providing an in-depth explanation of how the two-stage Delphi survey and structured literature review method were conducted, including qualitative and quantitative analyses. This article explains the search criteria and strategy that were applied to identify key publications referred to in the original manuscript. The current article further provides quantitative data from the two-stage Delphi survey that addresses the differences that exist in defining and managing asthma control.

## 3 Data Description

### 3.1 Literature review

A comprehensive literature search was performed. Search strings relating to the literature review are available in the Supplement of the original manuscript [1]. A PRISMA flow diagram detailing the number of manuscripts/congress abstracts included/excluded at each stage of the literature review is detailed in Figure 1 of the original manuscript. **Raw data file, ‘Asthma consensus DiB_Literature search_Raw data’ details the search strategy and identification of literature.** The below describes and explains the raw data file:

• The three sheets titled ‘PubMed_Search_History’, ‘Cochrane_Search History’, and ‘Embase_Search_History’ detail the search strings used, date of search, and associated number of results identified on PubMed, Cochrane, and Embase, respectively.
• From these searches, duplications were removed. A total of 558 manuscripts and 89 abstracts were screened, the details of these manuscripts are noted on sheets titled ‘Manuscripts’ and ‘Congress_Abstracts’, respectively. The following description of methodology and raw data files applies to both sheets:
  ○ Key words selected prior to the search, associated with ‘asthma’, ‘control’, ‘tests’, ‘measures’, ‘PROs’, and ‘Guidelines’ are detailed in the sheet titled ‘Keywords’. Appearance of any of these keywords was highlighted during the literature search, with results indicated by inclusion of a ‘Y’ under the appropriate category column (Columns J–0 in ‘Manuscripts’ and Columns I–N in ‘Congress_Abstracts’ sheets).
    • These keywords are also highlighted in the associated colours throughout the manuscript/abstract title and abstracts (Columns Q&R in ‘Manuscripts’ and Columns P&Q in ‘Congress_Abstracts’ sheets).
  ○ Once all 558 manuscripts and 89 abstracts were entered into the Excel sheet, Reviewer 1 screened the titles and abstracts of manuscripts/congress abstracts and noted in Column A whether the manuscript should be included for full data extraction, excluded, or if they were unsure. Column B was used to note a reason for exclusion, using a pre-defined drop-down option (all dropdown options are detailed in sheet titled ‘Exclusion reasons’. Column C was used to note additional notes by Reviewer 1.
  ○ All manuscripts/congress abstracts that were listed as ‘unsure’ were then reviewed by Reviewer 2, in addition to 20% of all other manuscripts/congress abstracts, to ensure consistency and agreement. Reviewer 2’s agreement for full text data extraction is noted in Column D.
  ○ GSK authors (AK, AKB, and SM) also did a final review of 20% of the manuscripts and noted their comments in Column F of sheet ‘Manuscripts’.
  ○ Final agreement to either include or exclude the titles in detail in Column G or ‘Manuscripts’ and Column F of ‘Congress_Abstracts’.
• A total of 243 manuscripts and 42 congress abstracts were included for full text review. **Screening and data extraction of the full texts is detailed in the Excel raw data file titled ‘Delphi data extraction_Raw data’**.
  ○ Of the proposed manuscripts for full text review and data extraction, 1 manuscript record could not be retrieved and was excluded from the data extraction.
  ○ Once data extraction was complete, the included manuscripts/congress abstracts were copied into the sheet titled ‘Included publications’, whilst the excluded manuscript/ congress abstracts were copied into the sheet titled ‘Excluded publications. Congress abstracts are highlighted with a blue cell in Row A and begin from Row 190 of ‘Included publications’ and Row 62 of ‘Excluded publications.
    • The full text of all publications was reviewed, and details were entered using either drop-down selections or free text (highlighted with ‘[Please select]’ or free text ‘[Free text]’ in Row 3, respectively). Drop down selections are provided in the sheet titled ‘Drop down selections’.
    • The full text reviewer went through each individual manuscript/congress, and where available, completed the data extraction template. If data or information weren't available, the cell was left blank.
    • Where it was decided at full text screening that a manuscript was not eligible for inclusion, a decision was made and noted in Row AJ of ‘Excluded publications’ along with an exclusion reason in Row AK.
• A total of 185 manuscripts and 31 abstracts were included in the literature review. Count data of included/excluded publications and exclusion reasons are detail in the ‘Overview’ sheet and feed into the PRISMA flow diagram (Figure 1 in the original manuscript).
• Counts of information identified during data extraction (e.g. if control was included as a study endpoint and if this was a primary, secondary, other endpoint) and the number of measures used to define/measure control are detailed in the sheet titled ‘Data analysis’.
  ○ The validated measures/guidelines identified as having been used in the literature, shown in sheet ‘Data analysis’, Row 31–52, Columns B&C, were then used to create the Table in sheet ‘Analysis of measures’.
    • Each measure/guideline was reviewed and identification of symptoms/therapy etc. used in each one was highlighted with a ‘tick’ symbol and used to create a Table.

### 3.2 Two-stage Delphi questionnaire

In addition to the comprehensive literature review, a two-stage Delphi survey of 82 panellists (73 HCPs and 9 authors) was included. Details on the development of the survey and the full questionnaires are available in the Online Repository of the original manuscript [1]. Here, we include raw data from the two rounds of the survey, including further information on analysis. **Please refer to raw data file, ‘Final Delphi Survey Responses_Raw Data’**.

• Data are presented as either percentages or counts in sheets titled ‘S1. Percentages’ or ‘S1. Counts’, respectively. In both sheets, Column B represents total values, Columns D–J stratify the results by current primary medical specialty, and Columns K–Z stratify by location. For the purposes of the below, we will refer to total values (Column B), throughout.
• Raw data for the screening questions (as per Survey 1 in the Online Supplement of the original manuscript [1]) are available as per the below:
  ○ Country of practice, first round survey screening Question 1: countries and count/percentage detailed in Columns A&B, Rows 8–15 for HCP respondents and Columns A&B, Rows 22–30 for author respondents.
  ○ Current primary medical specialty, first round survey screening Question 2: Rows 37–41 for HCP respondents and Columns A&B, Rows 48–52 for author respondents.
  ○ Years qualified in specialty, first round survey screening Question 3: Rows 59–65 for all respondents.
  ○ Percentage of time spent on given activities, first round survey screening Question 4: Rows 59–65 for all respondents: Mean values for all respondents presented in Rows 89 (% actively treating patients), 101 (% academic/research), and 113 (% admin/other).
  ○ Number of patients with asthma treated in a typical month (pre-COVID-19 pandemic), first round survey screening Question 5: Mean value for all respondents presented in Row 128.
  ○ Acceptance of terms and conditions and adverse event reporting, first round survey screening Question 6: Rows 137–138 (100% acceptance) and Rows 147–149, respectively.
• Raw data for responses given by all respondents (*n* = 82) in the first round survey (as per Survey 1 Online Supplement of the original manuscript [1]) are detailed in Table 1. All responses were available as quantitative data in sheets ‘S1. Percentages’ and ‘S1. Counts’. Where a free text response was requested, qualitative data were collated and detailed in sheet titled ‘S1. Open ended questions’. Note that where a question is open or provides opportunity for the respondent to provide free-text, the sheet ‘S1. Open ended questions’ should be used in the first instance as the quantitative data from the sheet ‘S1. Percentages’/’S1. Counts’ provides little information apart from that a response was provided by the responded. Where a response was given in non-English language, a simple online translation software was used to translate to English.

**Table 1:** Raw data file description for Survey 1 responses.

Survey question number	Raw data file sheet	Question asked (Column, Row)	Responses (Column, Row)
1 [Open question]*	‘S1. Percentages’ / ‘S1. Counts’	A, 152	B, 156
	‘S1. Open ended questions’		D
2 [Multi-response question]	‘S1. Percentages’ / ‘S1. Counts’	A, 159	B, 163–172
	‘S1. Open ended questions’		E, F
3 [Open question]*	‘S1. Percentages’ / ‘S1. Counts’	A, 175	B, 179
	‘S1. Open ended questions’		H
4 [Multi-response question]	‘S1. Percentages’ / ‘S1. Counts’	A, 182	B, 186–193
	‘S1. Open ended questions’		I
5 [Multi-response question]	‘S1. Percentages’ / ‘S1. Counts’	A, 196	B, 201–207
	‘S1. Open ended questions’		J
6a [Single-response question]	‘S1. Percentages’ / ‘S1. Counts’	B, 233	B, 235–242
6b [Single-response question]	‘S1. Percentages’ / ‘S1. Counts’	D, 233	D, 248–255
6c [Single-response question]	‘S1. Percentages’ / ‘S1. Counts’	F, 233	F, 235–241
6d [Single-response question]	‘S1. Percentages’ / ‘S1. Counts’	H, 233	H, 235–241
6e [Single-response question]	‘S1. Percentages’ / ‘S1. Counts’	J, 233	J, 235–241
6f [Single-response question]	‘S1. Percentages’ / ‘S1. Counts’	L, 233	L, 235–241
6g [Single-response question]	‘S1. Percentages’ / ‘S1. Counts’	N, 233	N, 235–241
6h [Single-response question]	‘S1. Percentages’ / ‘S1. Counts’	P, 233	P, 235–241
6i [Single-response question]	‘S1. Percentages’ / ‘S1. Counts’	R, 233	R, 235–241
6j [Single-response question]	‘S1. Percentages’ / ‘S1. Counts’	T, 233	T, 235–241
6k [Single-response question]	‘S1. Percentages’ / ‘S1. Counts’	V, 233	V, 235–241
6l [Single-response question]	‘S1. Percentages’ / ‘S1. Counts’	X, 233	X, 235–241
6m [Single-response question]	‘S1. Percentages’ / ‘S1. Counts’	Z, 233	Z, 235–241
6n [Single-response question]	‘S1. Percentages’ / ‘S1. Counts’	AB, 233	AB, 235–241
7 [Open-question]*	‘S1. Percentages’ / ‘S1. Counts’	A, 401	B, 405
	‘S1. Open ended questions’		L

First round survey responses were recorded as qualitative data in sheets ‘S1. Percentages’ and ‘S1. Counts’. For free text responses, qualitative data were collated and detailed in sheet titled ‘S1. Open ended questions’. *Note that where a question is open or provides opportunity for the respondent to provide free-text, the sheet ‘S1. Open ended questions’ were used in the first instance as the quantitative data from the sheet ‘S1. Percentages’/’S1. Counts’ provides little information apart from that a response was provided by the respondent.

• Questions for Survey 2 were developed using collated free text responses given in Survey 1
  ○ For Survey 1, Question 6, where ‘NET Agree’ (sheets ‘S1. Percentages’ and ‘S1. Counts’, Column A, Row 241) or ‘NET Disagree’ (sheets ‘S1. Percentages’ and ‘S1. Counts’, Column A, Row 240) were ≥ 66%, agreement was assumed and the questions were not explored further in Survey 2.
• Raw data for responses given by all respondents (*n* = 82) in the second-round questionnaires (as per Survey 2 Online Supplement of the original manuscript [1]) are detailed in Table 2.
  ○ Note that for Question 2, no ‘other’ responses were given, and therefore no free text responses were provided.

**Table 2:** Raw data file description for Survey 2 responses.

Survey question number	Raw data file sheet	Question asked (Column, Row)	Responses (Column, Row)
1a [Single-response question]	‘S2. Percentages’ / ‘S2. Counts’	B, 91	B, 92–99
1b [Single-response question]	‘S2. Percentages’ / ‘S2. Counts’	D, 91	D, 92–99
1c [Single-response question]	‘S2. Percentages’ / ‘S2. Counts’	F, 91	F, 92–99
1d [Single-response question]	‘S2. Percentages’ / ‘S2. Counts’	H, 91	H, 92–99
1e [Single-response question]	‘S2. Percentages’ / ‘S2. Counts’	J, 91	J, 92–99
1f [Single-response question]	‘S2. Percentages’ / ‘S2. Counts’	L, 91	L, 92–99
1g [Single-response question]	‘S2. Percentages’ / ‘S2. Counts’	N, 91	N, 92–99
1h [Single-response question]	‘S2. Percentages’ / ‘S2. Counts’	P, 91	P, 92–99
2 [Multi-response question]	‘S2. Percentages’ / ‘S2. Counts’	A, 102	B, 106–112
3a [Single-response question]	‘S2. Percentages’ / ‘S2. Counts’	B, 134	B, 135–148
3b [Single-response question]	‘S2. Percentages’ / ‘S2. Counts’	D, 134	D, 135–148
3c [Single-response question]	‘S2. Percentages’ / ‘S2. Counts’	F, 134	F, 135–148
3d [Single-response question]	‘S2. Percentages’ / ‘S2. Counts’	H, 134	H, 135–148
3e [Single-response question]	‘S2. Percentages’ / ‘S2. Counts’	J, 134	J, 135–148
3f [Single-response question]	‘S2. Percentages’ / ‘S2. Counts’	L, 134	L, 135–148
3g [Single-response question]	‘S2. Percentages’ / ‘S2. Counts’	N, 134	N, 135–148
3h [Single-response question]	‘S2. Percentages’ / ‘S2. Counts’	P, 134	P, 135–148
3i [Single-response question]	‘S2. Percentages’ / ‘S2. Counts’	R, 134	R, 135–148
4a [Single-response question]	‘S2. Percentages’ / ‘S2. Counts’	B, 165	B, 166–167
4b [Single-response question]	‘S2. Percentages’ / ‘S2. Counts’	D, 165	D, 166–167
4c [Single-response question]	‘S2. Percentages’ / ‘S2. Counts’	F, 165	F, 166–167
4d [Single-response question]	‘S2. Percentages’ / ‘S2. Counts’	H, 165	H, 166–167
4e [Single-response question]	‘S2. Percentages’ / ‘S2. Counts’	J, 165	J, 166–167
4f [Single-response question]	‘S2. Percentages’ / ‘S2. Counts’	L, 165	L, 166–167
5 [single-response question]	‘S2. Percentages’ / ‘S2. Counts’	A, 177	B, 181–189
6 [single-response question]	‘S2. Percentages’ / ‘S2. Counts’	A, 192	B, 196–208
• Once both surveys were complete, analysis of the qualitative responses given in Questions 1, 3 and 7 of Survey 1 were performed, **please refer to sheet ‘Questions and tallies’ in the‘raw data file, ‘Final Delphi Survey Responses_Raw Data’**.
  ○ The reviewer identified key themes and categorized the responses given according to the themes (Columns F–H for Question 1; O–S for Question 3; and Column X for Question 7).
  ○ Responses within these categories were counted and are detailed in sheet titled ‘Count and analysis’ (Rows 1–12 for Question 1; Rows 27–35 for Question 3; and Rows 38–50 for Question 7).
  ○ Guidelines mentioned as an additional open text response by questionnaire respondents in Question 2 were also counted and tallied (Rows 14–25).
  ○ Specific values pertaining to ACT, ACQ, and PFT values respondents considered indicative of ‘good’ control were counted and detailed:
    • ACT: analysis noted in ‘Questions and tallies’ Column Q, tally noted in ‘Count and analysis’ Columns E&F, Rows 27–36.
    • ACQ: analysis noted in ‘Questions and tallies’ Column R, tally noted in ‘Count and analysis’ Columns H&I, Rows 27–31.
    • PFT: analysis noted in ‘Questions and tallies’ Column S, tally noted in ‘Count and analysis’ Columns K&L, Rows 27–31.

## 4 Experimental Design, Materials and Methods

Using a two-way comparative approach, we sought to collate qualitative insight data obtained from a two-stage Delphi survey and compare it with results from a structured literature review, with the aim to reach expert consensus on challenges around asthma control in clinical practice. By definition, a Delphi survey is a systematic technique that aims to reach a consensus over a disputed topic [2]. The first part of the present study was performed as two sequential Delphi questionnaires shared with, and completed by, practicing HCPs to reach expert consensus on definitions of asthma control used in clinical practice, and to identify and potentially alleviate disparities in asthma management between patients and HCPs [1]. The second part of the study utilized quantitative data from the structured literature review to evaluate measures of asthma control used in clinical trials and studies. Both the Delphi survey and literature review were carried out by a research team at Ashfield MedComms, an Inizio company, funded by GSK.

## 5 Two-Stage Delphi Survey Technique

### 5.1 Screening criteria for panelists

A third-party, centralized database (Sermo) was used to invite practicing HCPs to provide further expert opinion to the nine practicing clinician study authors (GWC, AS, LPdL, CDR, JDB, GG, HI, MD, and DY). A total of 2350 panelists were invited. Following screening questions relevant to their appropriate specialty (pulmonologists, allergists, and general practitioners), number of years of experience within their specialty (> 3 years), and the proportion of time actively treating patients (≥ 40%), 73 eligible practicing HCPs from seven different countries split based on the locations of practice for the nine authors (Argentina, Australia, Brazil, China, Italy, Japan, and Spain) agreed to participate.

Panelists were requested to complete the survey within 1 week, and would only be eligible to proceed to completing the subsequently developed second-round questionnaire if they had completed the first-round. Panelists who did not respond (*n* = 19) to the second-round survey were followed up with by a member of the research team.

### 5.2 First-round survey development

In this study, the Delphi technique used two questionnaires. The first to gain HCP insights and opinions, and the second to build upon responses in the first stage and try to reach consensus. The first questionnaire was developed with input from all authors who also reviewed and approved the final disseminated questionnaire. Both rounds of surveys were shared with survey respondents in March and April 2021, respectively. The first questionnaire consisted of seven open- and closed-, single- and multiple-choice, and Likert-scale based questionnaire and was shared with, and completed by, 82 panelists, including the nine authors Once results were received and collated, those gained from the Likert-scale questions were categorized as disagree, neither agree nor disagree, and agree, to quantitate a consensus, which was determined as reached when ≥ 66% of panelists voted within the agree or disagree category. Panelists’ responses to open-ended questions were collated to gather ideas and qualitative comments, which were translated into a quantitative outputs where possible or sorted into themes appropriate to the question, which were analyzed in greater detail during the second-round questionnaire to reach a consensus.

### 5.3 Second-round survey

The second-round survey was used to further explore qualitative responses provided by panelists during the first-round questionnaire and provide closed- or Likert-response questions to try and reach consensus. Where consensus had not been achieved in the first round, questions were re-evaluated, rephrased or restructured where appropriate, and shared with the panelists (*n* = 63) who completed the first questionnaire within the specified 1-week timeframe. In this round, there were a total of six closed questions to gain a consensus. Due to regulatory-imposed constraints, patients were not invited to participate in this study and therefore all suggestions of patient opinion or experience relating to asthma control are HCP-perceived and -reported.

## 6 Structured Literature Review

The second part of our study used results from a structured literature review to identify measures and guidelines for asthma control in clinical publications. The results were compared with those gained from the Delphi study. Literature published between 2004 (publication of the study by Bateman *et al*, which provided insights into asthma control [3]) and the date of the search (March 2021) were identified. PubMed, Cochrane, and Embase databases were searched for relevant articles and congress abstracts; only abstracts published from 2018–2021 were included due to the assumption that those published prior would likely be available as full, peer-reviewed manuscripts. Reviewers from the research team at Ashfield MedComms, an Inizio company, used criterion which was pre-defined and approved by all authors to manually screen retrieved manuscript titles and abstracts. Only studies in adults (aged ≥ 18 years), with asthma control as a study endpoint, and an appropriate study design (randomized controlled trial, systematic review, meta-analysis, and/or real-world evidence) were suggested as suitable for full text retrieval and review.

Of the results proposed as suitable for full text retrieval and review, 20% (randomly selected using a random number generator) were screened by a second reviewer in order to ensure alignment and agreement with the inclusion criteria. In case of disagreement, a third reviewer from the research team reviewed and discussed with the other reviewers, until a final decision was met. Three authors (SM, AK, and AKB) reviewed 10% (randomly generated) of all proposed included and excluded publications to further ensure that agreement was reached. The structured literature review results were provided in an accessible format to all study authors to ensure opportunity for review and comments. If agreement was still not reached after reviewing study titles and abstracts, the articles were included for full data extraction, at which point a final inclusion/exclusion decision could be made by the study authors. From the included publications, results were quantified and stratified according to: (1) trial design; (2) control as primary/secondary endpoint; (3) population; (4) total number of patients included; (5) center type; (6) country; (7) measure of control used (guidelines, validated measure, symptomatic measure of control [e.g., frequency of exacerbations, use of rescue medication], patient-reported measures of control [e.g., sleep disturbances, impact on day-to-day activities]); and (8) any further details. Results of the structured literature review were descriptive only; no assumptions were made prior to the research being conducted or during data extraction and data/study results were extracted and recorded verbatim.

## Ethics Statements

This research complies with UK Data Protection Act (GDPR) and with the British Healthcare Business Intelligence Association's (BHBIA) Legal & Ethical Guidelines, along with the European Pharmaceutical Market Research Association's (EphMRA).

All participants agreed to answering the questionnaire anonymously, with no personal data or any type of information linking the answers to a specific participant.

## CRediT Author Statement

All authors were involved in the study conception, data acquisition, and data analysis and/or interpretation and in writing/critical review if draft versions of this article and approval of the final version to be submitted for publication.

Funding: This work was supported by GSK.

## Declaration of Competing Interest

The authors declare the following financial interests/personal relationships which may be considered as potential competing interests: A. Spanevello reports consulting fees from GSK; payment or honoraria from AstraZeneca, Chiesi, and GSK; and participation on a data safety monitoring board or advisory board for GSK. L. Pérez de Llano reports grants or contracts from AstraZeneca, Esteve, FAES Pharma, and Teva, consulting fees from AstraZeneca, GEBRO, Gilead, GSK, MSD, Novartis, and Sanofi; payment or honoraria from AstraZeneca, Chiesi, Esteve, GSK, LEO Pharma, MSD, Novartis, and Sanofi; support for attending meetings and/or travel from AstraZeneca, Chiesi, FAES Pharma, GSK, and Novartis; and participation on a data safety monitoring board or advisory board for AstraZeneca. C. Domingo Ribas has received funding for travel or speaker fees from ALK, Almirall, AstraZeneca, Boehringer Ingelheim, Chiesi, Esteve, Ferrer, GSK, Menarini, Novartis, Pfizer, and Stallergenes, and declares no specific conflicts of interest to report regarding this paper. J.D. Blakey reports grants or contracts from AstraZeneca, GSK, and Novartis; consulting fees from Boehringer Ingelheim, Chiesi, and GSK; payment or honoraria from AstraZeneca, Chiesi, and GSK; support for attending meetings and/or travel from AstraZeneca, Boehringer Ingelheim, and GSK; receipt of medical writing support from GSK and Teva; payment to their institution for advisory work from Asthma Australia; and unpaid advisory work from Asthma WA. H. Inoue declares research grants from Boehringer Ingelheim, payment or honoraria for lectures and advisory committees from AstraZeneca, Boehringer Ingelheim, GSK, Kyorin, Novartis, and Sanofi. G.W. Canonica declares no conflict of interest for the current paper, and reports having received research grants as well as being lecturer or having received advisory board fees from A.Menarini, Allergy Therapeutics, Anallergo, AstraZeneca, Chiesi, FAES Pharma, Firma, Genentech, GSK, Guidotti-Malesci, Hal Allergy, Innovacaremd, Novartis, OmPharma, RedMaple, Sanofi-Aventis, Sanofi-Genzyme, Stallergenes-Greer, ThermoFisher, Uriach Pharma, and Valeas. D. Yang, and M. Dalcolmo declare that they have no disclosures of interest. G. Garcia declares participation in advisory boards for AstraZeneca, Boehringer Ingelheim, GSK, Novartis, and Sanofi; participation in sponsored activities for AstraZeneca, Boehringer Ingelheim, GSK, Novartis, Sanofi, and Phoenix; principal investigator for AstraZeneca, Boehringer Ingelheim, Chiesi, Covance, GSK, Iqvia, Novartis, Parexel, PPD, Sanofi, and Zambon. S. Mokashi, A. Kurne, and A. Butta were GSK employees at the time of study conduct and hold shares in GSK.

## Data Availability

Asthma control conundrum in clinical practice – Data from a two-stage Delphi survey and literature review (Original data) (Zenodo).
